# ROUND LIGAMENT REPAIR OF THE BILE DUCT AS TREATMENT OF BILE DUCT INJURIES: CASE REPORT

**DOI:** 10.1590/0102-672020180001e1443

**Published:** 2019-08-26

**Authors:** Juan Alvarado IRIGOYEN, Hernán Herrera CORTES, Andrés Troncoso TRUJILLO, Héctor Losada MORALES, Jorge Silva ABARCA, Luis Acencio BARRIENTOS, Oriel Arias ROVIRA, Samuel Zúñiga RIVILLO

**Affiliations:** 1 Departamento de Cirugía, Universidad de la Frontera;; 2 Servicio de Cirugía, Universidad de La Frontera;; 3 Servicio de Cirugía, Clinica Alemana Temuco, Chile.

**Keywords:** Bile ducts injuries, Round ligament, Cholecystitis, Cholecystectomy, Lesões das vias biliares, Ligamento redondo, Colecistite, Colecistectomia

## INTRODUCTION

Bile duct injuries have various etiologies^2^ ^,^ ^6^. For their repair, there are several surgical techniques adjusted to the clinical situation of each patient^9^ ^,^ ^10^. In some common bile duct injuries round ligament repair has been suggested as an alternative to a possible biliodigestive derivation. However, the evidence with respect to its generalized use is limited^1^ ^,^ ^3^ ^,^ ^8^.

We present the case of a patient operated for acute cholecystitis with a bile duct injury associated with necrosis of the common bile duct that was repaired with a round ligament patch.

## CASE REPORT

Male, 68 years old, history of non-insulin-dependent diabetes mellitus type 2, with four days of evolution of abdominal pain in right hypochondrium and fever, no jaundice. Abdominal ultrasound showed overdistended gallbladder with diffuse thickening, 17 mm hepatocholedochus with some echoes and thin walls in its interior. Evaluated by the emergency service, it was decided to try medical treatment with analgesics, antipyretics and antibiotics.

**FIGURE 1:**
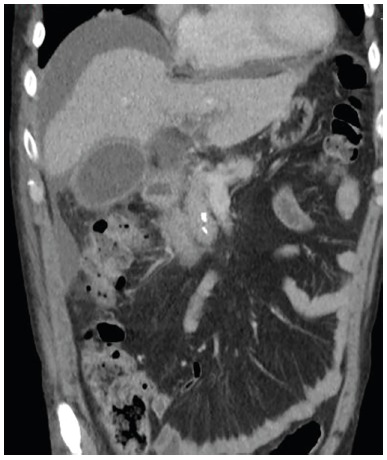
Preoperative abdominal CT scan: free fluid can be seen in the right subphrenic and subhepatic areas, with the distended vesicle and perivesicular edema that extends to the hepatoduodenal ligament.

On the second postoperative day, the patient developed a fever, positive Murphy’s sign, cholestatic pattern and elevated inflammatory markers. Control CT was done that revealed free intra-abdominal fluid associated with perivesicular inflammatory changes (Figure 1).

Given that the patient did not respond adequately to the medical treatment, surgical treatment was decided. He was brought to the operating room for an exploratory laparotomy, where a right subphrenic collection, a gallbladder mass with necrotic vesicle, and a 12 mm choledochus with wall necrosis at the level of the carina on its necrotic previous side were identified.

An open cholecystectomy was performed; choledochotomy showed no evidence of lithiasis, only detritus in the bile duct.

**FIGURE 2:**
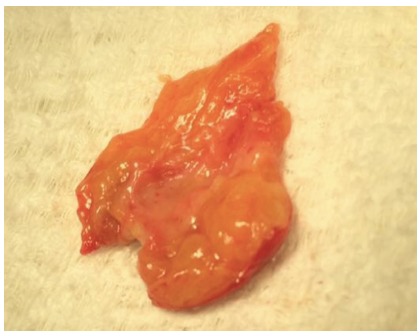
Round ligament patch: The round ligament has been divided and dissected from adjacent adipose tissue. Longitudinal opening has been made to give it the rectangular shape of the patch.

In view of the injury on the anterior side of the choledochus and the fragility of the tissue, the defect was repaired with a round ligament patch at the edges of the injury. The round ligament was divided and dissected, and a longitudinal incision was made to reach the rectangular configuration of the patch (Figure 2). 

It was sutured with PDS 4-0, putting the endoluminal surface of the ligament into contact with the choledochus lumen. The surgery was completed with the installation of a Kehr tube number 14.

**FIGURE 3:**
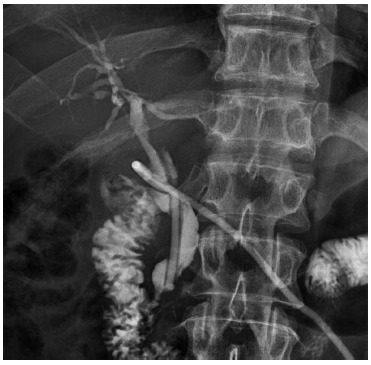
T-tube cholangiography performed at six weeks post-repair: the Kehr tube is visualized in situ, with no evidence of bile duct stenosis, with good passage of the contrast medium to the duodenum.

The patient completed his postoperative period favorably, without complications, and was discharged on the tenth day. A T-tube cholangiography was performed at six weeks (Figure 3), with no evidence of filtration or stenosis; so, the Kehr tube was removed.

**FIGURE 4:**
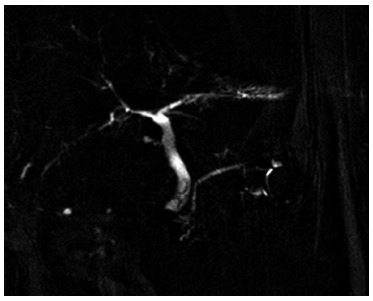
Follow-up NMR performed at one year post-repair: fine intrahepatic bile duct can be seen and there is no evidence of stenosis at repair level

An annual follow-up with magnetic cholangioresonance was done (Figure 4), where the intrahepatic bile duct was found without significant expansion, and the proximal extrahepatic bile duct appeared normal with no evidence of stenosis.

The patient has been asymptomatic in subsequent check-ups, and follow-up liver tests also having been normal.

## DISCUSSION

Common bile duct injuries are well characterized and there are various treatment options. However, in this patient, the major flaw made evident intra-operatively was the fragility of the tissue, and the risk of postoperative stenosis, which did not allow for either a primary repair or a biliodigestive derivation.

There are few case reports in the literature on the use of this technique for the repair of the common bile duct. Most indicate round ligament flaps^1^ ^,^ ^3^ ^,^ ^8^, which are not the same as the technique presented in this case. The use of a round ligament patch impresses as being a safe and feasible technique to perform on a select group of patients.

In our hospital this patch has been used to treat three bile duct injuries (two iatrogenic and this one associated with necrosis of the choledochus), with all being successful, and follow-ups longer than two years that demonstrate an adequate quality of life and absence of stenosis.

Treatment of bile duct injuries is complex and the range of possibilities goes from endoscopic therapy for partial injuries to reconstruction with a Roux-en-Yhepaticojejunal anastomosis for complex injuries. In our team the associated vascular damage has always been investigated^4^ ^,^ ^5^ ^,^ ^7^.

However, in patients with partial bile duct defects (non-linear), where inflammation and sepsis are involved either due to the underlying disease as with this patient or the biliperitoneum associated with iatrogenic injuries of the bile duct, a reconstruction of the bile duct with a hepaticojejunal anastomosis is very complex and the inflammation around the bile duct has been considered an adverse factor in the prognosis of these patients. One option for such patients is to install a T-tube and to make the repair with a round ligament patch.

The technical considerations must include the dissection of the adipose tissue that surrounds the round ligament and its longitudinal opening so that it acquires the rectangular shape of the patch.

The patch can be affixed to the bile duct if the fragility and inflammation of the tissue allows it or to the pericholedocian tissue. If the bile duct is dissected, the patch can be placed circumferentially, ensuring that it is affixed so it does not move.

In some patients the Kehr tube has been placed through the defect, and the patch has been used to cover this defect. We consider the use of magnification important and we affix the patch with prolene or PDS. We always leave a subhepatic drain to treat a possible biliary fistula.

We consider the technique described to be safe, reproducible and can be incorporated into the tools to treat bile duct injuries.
